# Research on Microstructure, Mechanical Properties, and High-Temperature Stability of Hot-Rolled Tungsten Hafnium Alloy

**DOI:** 10.3390/ma17153663

**Published:** 2024-07-24

**Authors:** Yi Yin, Tiejun Wang, Sigui Qin, Wanjing Wang, Yingli Shi, Hongxin Yu

**Affiliations:** 1 Central lron & Steel Research Institute, Beijing 100081, China; yinyi230401@163.com; 2 Advanced Technology & Materials Co., Ltd., Beijing 100081, China; qinsigui@atmcn.com (S.Q.); shiyingli@atmcn.com (Y.S.); yuhongxin@atmcn.com (H.Y.); 3 Institute of Plasma Physics, Hefei Institutes of Physical Science, Chinese Academy of Sciences, Hefei 230031, China; wjwang@ipp.ac.cn; 4 University of Science and Technology of China, Hefei 230026, China

**Keywords:** plasma-facing materials, hot rolling, dispersion strengthening, precipitation strength

## Abstract

W-(0, 0.1, 0.3, 0.5) wt.% Hf (mass fraction, wt.%) materials were fabricated by the powder metallurgy method and hot rolling. The microstructure, mechanical properties, and high-temperature stability of alloys with varying compositions were systematically studied. The active element Hf can react with the impurity O segregated at the grain boundary to form fine dispersed HfO_2_ particles, refining the grains and purifies and strengthening the grain boundary. The average size of the sub-grains in the W-0.3 wt.% Hf alloy is 4.32 μm, and the number density of the in situ-formed second phase is 6.4 × 10^17^ m^−3^. The W-0.3 wt.% Hf alloy has excellent mechanical properties in all compositions of alloys. The ultimate tensile strength (UTS) is 1048 ± 17.02 MPa at 100 °C, the ductile fracture occurs at 150 °C, and the total elongation (TE) is 5.91 ± 0.41%. The UTS of the tensile test at 500 °C is 614 ± 7.55 MPa, and the elongation is as high as 43.77 ± 1.54%. However, more Hf addition will increase the size of the second-phase particles and reduce the number density of the second-phase particles, resulting in a decrease in the mechanical properties of the tungsten alloy. The isochronal annealing test shows that the recrystallization temperature of W-Hf alloy is 1400 °C, which is 200 °C higher than rolling pure tungsten.

## 1. Introduction

The tokamak device uses a magnetic field to constrain high-temperature and high-density plasma to achieve controllable thermonuclear fusion. Plasma-facing materials (PFMs) are key factors restricting its development. Tungsten has attracted extensive attention due to its excellent properties such as a high melting point (3540 °C), sputtering resistance, low tritium retention rate, and low thermal expansion coefficient, and is considered to be one of the most promising candidate materials for plasma-facing materials in fusion reactors [1,2,3]. However, tungsten exhibits severe brittleness in a variety of states, including low-temperature brittleness, irradiation brittleness, and recrystallization brittleness, which seriously restricts its wide application as PFM [4,5,6,7]. As a metal with a body-centered cubic structure, W has few slip systems, low dislocation mobility, and intrinsic brittleness [8]. In addition, oxygen, nitrogen, phosphorus, and other elements are easy to segregate impurities at the grain boundaries and weaken the grain boundary, which is considered to be one of the main reasons for the intergranular fracture of tungsten and has a significant effect on the fracture toughness of tungsten [9,10].

Over the past few decades, researchers have been working to improve the ductility of tungsten and reduce ductile–brittle transition temperature (DBTT). Increasing the grain boundary area by refining the grains can reduce the average concentration of impurities at the grain boundaries and improve the toughness of tungsten [11]. At the same time, the grain boundaries can be used as a defect trap to attract point defects caused by irradiation and inhibit the growth of helium bubbles [12,13,14,15]. Therefore, manufacturing fine-grained tungsten and eliminating the influence of grain boundary impurities seem to be very meaningful for improving the performance of tungsten. However, fine-grained pure tungsten is usually unstable, and the grains grow rapidly at high temperatures, making it difficult to manufacture and limiting high-temperature applications. Grain Boundary Segregation Engineering (GBSE) can enhance the cohesion of W grain boundaries by adding small amounts of active elements such as Ti, Zr, Hf, and rare earth elements such as La and Y. Through heat treatment and mechanical mixing, these elements segregate at the grain boundaries, strengthen the grain boundaries, and reduce the segregation of impurity elements at the grain boundaries [16,17]. In recent years, the development of ab initio simulations such as the density functional theory can help to identify the grain boundaries strengthening elements of a given matrix material. The cohesive effect on the grain boundaries of transition metals such as Hf, Zr, and Re can significantly strengthen W grain boundaries [18,19]. At the same time, the use of a small number of oxide nanoparticles dispersed in tungsten, such as La_2_O_3_, Y_2_O_3_, and ZrO_2_ [20,21,22], can effectively inhibit grain growth at high temperature, stabilize microstructure, improve high-temperature strength, recrystallization temperature, and greatly improve the creep resistance of tungsten [23]. For brittle metal W with bcc structure, thermomechanical processing such as rolling and forging can eliminate internal pores, increase dislocation density and reduce the DBTT of W [24,25]. 

In this work, W-(0, 0.1, 0.3, 0.5) wt.% Hf alloys were manufactured by powder metallurgy process. In order to understand the influence of different Hf doping contents on the microstructure, mechanical properties, and high thermal stability of the alloy, a high temperature tensile test and an isochronal annealing test were carried out. In addition, the chemical composition, microstructure, and distribution of the in situ second phase were also characterized. The results show that the introduction of Hf improves the high temperature strength and low temperature toughness of pure tungsten and increases the recrystallization temperature of pure tungsten. Starting from the microstructure, the internal mechanism of Hf strengthening W is analyzed in detail.

## 2. Materials and Methods

### 2.1. Materials Preparation

Pure W (PW) samples and W-(0.1, 0.3, 0.5) wt.% Hf sheets were prepared from pure W powder (average particle size 2.8 μm, purity ≥ 99.99%) and HfH_2_ powder (average particle size 1–3 μm, purity ≥ 99%). The mixed powder weighed according to the mass ratio was milled in a planetary-type ball mill in an argon atmosphere for 4 h. The ball-to-powder weight ratio was 8:1, and the rotation speed was 300 rpm. Tungsten carbide balls and mortars were used to minimize possible impurity pollution. The mixed powder was initially consolidated by cold isostatic pressing technology. After the pressure was 200 MPa and the holding time was 5 min, a green body with a size of about 175 mm × 95 mm × 48 mm was obtained. Then, the green body was kept in a hydrogen atmosphere at 2300 °C for 7 h to obtain a sintered sample with a size of about 145 mm × 80 mm × 40 mm. Subsequently, after five passes of rolling in a hydrogen atmosphere at 1400–1600 °C, the deformations of each pass were 15%, 15%, 20%, 25%, and 25%, respectively. Finally, all rolled plates with thicknesses of about 13 mm were prepared, and the total deformation was about 68%. RD (rolling direction), TD (transverse direction), and ND (normal direction) were used to represent the orientation of the rolled plates. All samples were annealed at 1100 °C for 1 h in a hydrogen atmosphere to release the residual stress caused by machining.

### 2.2. Mechanical Property Tests and Microstructure Observation

The Archimedes drainage method was used to measure the true density of the sample [26]. 

The Vickers hardness of the samples in the original rolling state and after annealing at different temperatures was measured. The measurements were performed on the RD-TD surfaces and TD-ND surfaces of the sample, respectively. The loading force was 30 kgf and the holding time was 13 s. A total of 6 repeated measurements were performed on the same sample surface along a preset straight-line trajectory, and the distance between the two adjacent indentation centers was at least 3 times the length of the indentation diagonal.

The high temperature tensile test was carried out on an Instron-5967 electronic universal material testing machine in a vacuum environment. The tensile specimen is round rod-shaped and the working length along rolling direction is 25 mm, test temperatures were 100 °C, 150 °C, 200 °C, 300 °C, 400 °C, and 500 °C, and the displacement rate was 0.05 mm/min. In order to ensure the repeatability of the experiment, each mechanical property test was carried out at least three times.

The high-temperature thermal stability of alloys was evaluated by annealing experiments at 1200–1600 °C for 1 h. Annealing was carried out in a vacuum high-temperature resistance furnace with a vacuum degree of 1 × 10^−3^ Pa and a heating rate of 10 °C/min. Samples were heated to a set temperature and cooled with the furnace after holding for 1 h.

The microstructure and fracture morphology of the specimens at different stages were observed by scanning electron microscope (SEM, JSM 7200F, JEOL Ltd., Tokyo, Japan) equipped with EDS (Oxford X-Max, Oxford, UK). Electron backscatter diffraction (EBSD) was performed on an Oxford Nordlys Max2 (Oxford, UK) device connected to a JSM 7200F SEM. The acceleration voltage is 20 kV. Through AZtecCrystal software (Version 3.0) processing. The low-angle grain boundary (LAGB) and high-angle grain boundary (HAGB) are defined as grain boundary misorientations of 2°–15° and above 15°, respectively. The EBSD image was slightly filtered to eliminate the influence of the second-phase particles on the observation of tungsten grains. Before the EBSD test, all samples were electropolished in 5% NaOH solution at a constant voltage of 11 V and a corresponding current density of 3 mA/mm^2^. The microstructure of the second phase and tungsten matrix was detected by high resolution transmission electron microscope (TEM, FEI Tecnai G2 F20, Thermo Fisher Scientific Inc., Waltham, MA, USA) equipped with Bruker, Billerica, MA, USA, XFlash 5030 EDS. The sample for TEM needed to be mechanically thinned by sandpaper, ground to a thickness of less than 100 μm, and then precisely cut into a wafer with a diameter of 3 mm. Subsequently, the wafer was initially thinned accurately using a pit instrument to form one or more pits on the surface of the sample. The final thinning was carried out using a focused ion beam until a thin zone appeared in the center of the sample.

## 3. Results

### 3.1. Basic Properties of the PW and W-Hf Materials

Table 1 summarizes the relative density and Vickers hardness of PW and W-Hf alloys with different compositions. The results show that the relative density of the four materials is greater than 99%, which is because the hot rolling process can effectively eliminate the pores in the sintered material. Among them, the relative density of the W-0.3% Hf sample is the highest, reaching 99.43 ± 0.03%, and the microhardness is the highest (RD-TD surface HV_30_ = 481.47 ± 4.82). As the Hf content increases to 0.5%, the relative density and hardness of the material decrease slightly. This phenomenon is because excessive Hf is easy to accumulate at the grain boundary, which reduces the binding energy of W grain boundary and increases the appearance of pores, so that stress concentration is more likely to occur, resulting in the decrease in mechanical properties [27].

### 3.2. Microstructure of the PW and W-Hf Materials

**Figure 1:**
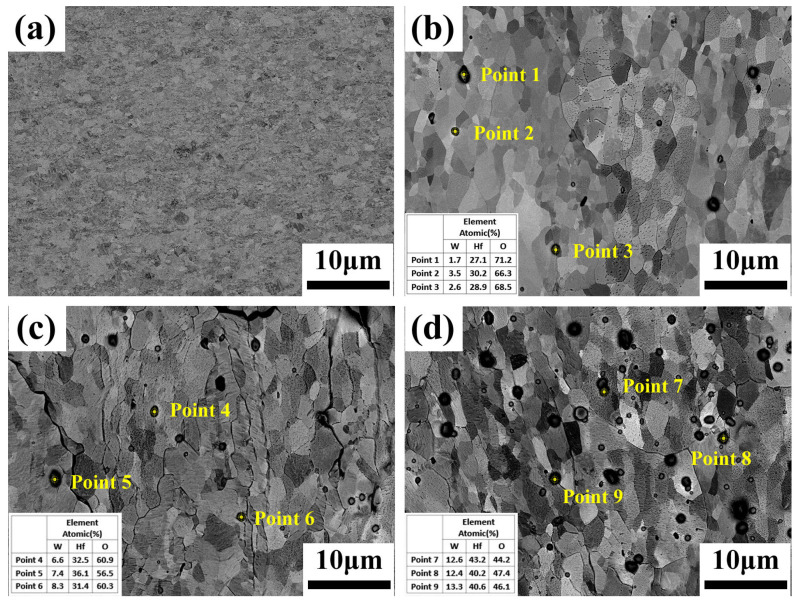
BSE-SEM images of the RD-TD face of the rolling PW (**a**) and W-Hf alloys and corresponding EDS. (**b**) W-0.1% Hf; (**c**) W-0.3% Hf; (**d**) W-0.5% Hf.

The high-magnification BSE-SEM images of the RD-TD surface of PW and W-Hf alloys are shown in Figure 1. The analysis shows that due to the dynamic recrystallization of W-Hf alloy during hot rolling, the fine tungsten grains basically have an equiaxed structure. Most of the black second phase is distributed in the grain matrix, and a small part is segregated at the grain boundary. The elemental analysis of W-0.1% Hf, W-0.3% Hf, and W-0.5% Hf random black particles was carried out by EDS. The results show that the second phase is only composed of W, Hf, and O elements. At the same time, the average atomic ratios of W:Hf:O in the second phase of W-0.1% Hf, W-0.3% Hf, and W-0.5% Hf samples are 2.6:28.7:68.7, 7.4:33.3:59.3, and 12.7:41.3:46.0, respectively. The results show that the content of W and Hf in the second phase increases slightly and the content of O decreases after removing the background effect. Since Hf is an active metal with a strong affinity for oxygen, an amount of dehydrogenated Hf can be completely oxidized by trace oxygen in tungsten to form oxides [28]. The detailed analysis of the formation process of the second phase will be elaborated in the next section.

**Figure 2:**
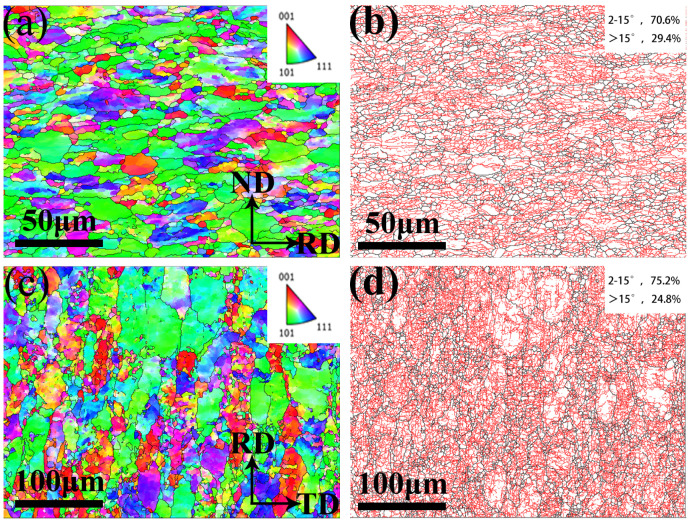
EBSD of W-0.3% Hf samples. (**a**) EBSD orientation mapping of RD-ND surface. (**b**) Distribution of high-angel grain boundary (black) and low-angel grain boundary (red) of the RD-ND surface. (**c**) EBSD orientation mapping of RD-TD surface. (**d**) Distribution of high-angel grain boundary (black) and low-angel grain boundary (red) of the RD-TD surface.

Figure 2 displays the EBSD analysis result of RD-ND and RD-TD surface of W-0.3% Hf alloy. The definitions of the colors and directions are as follows: red for <001>, green for <101>, and blue for <111>. Figure 2a,c show that some tungsten grains in the alloy have [101] grain orientation parallel to the rolling direction. The tungsten grains with larger particle size (grains with misorientation angle, θ > 15°, represented by the black line of grain boundary in Figure 2b,d are elongated along the RD direction. The average length and width of W grains on the RD-ND surfaces of PW, W-0.1% Hf, W-0.3% Hf, and W-0.5% Hf samples were 63.8 μm/15.6 μm, 31.2 μm/8.6 μm, 18.8 μm/4.4 μm, and 25.1 μm/6.5 μm, respectively, and the average aspect ratio of the grains is about 4:1, which is not much different. The grains contain equiaxed sub-grains (sub-grains with misorientation angle, 2° < θ < 15°, represented by grain boundary red lines in Figure 2b,d). The average sizes of more than 4000 equiaxed sub-grains on the RD-ND surface of PW, W-0.1% Hf, W-0.3% Hf, and W-0.5% Hf samples were 13.06 μm, 6.72 μm, 4.32 μm, 6.22 μm, respectively. It is precisely because of the presence of sub-grains that the low-angle grain boundaries account for a large proportion, and the orientation difference results deviate from the random distribution curve. This structure of elongated parent crystal composed of fine equiaxed sub-grains not only weakens the anisotropy of the plate but also facilitates the synergistic improvement of material strength and toughness: the elongated parent crystal has good ductility, and the fine equiaxed sub-grains provides more grain boundaries to hinder dislocation movement and improve material strength.

**Figure 3:**
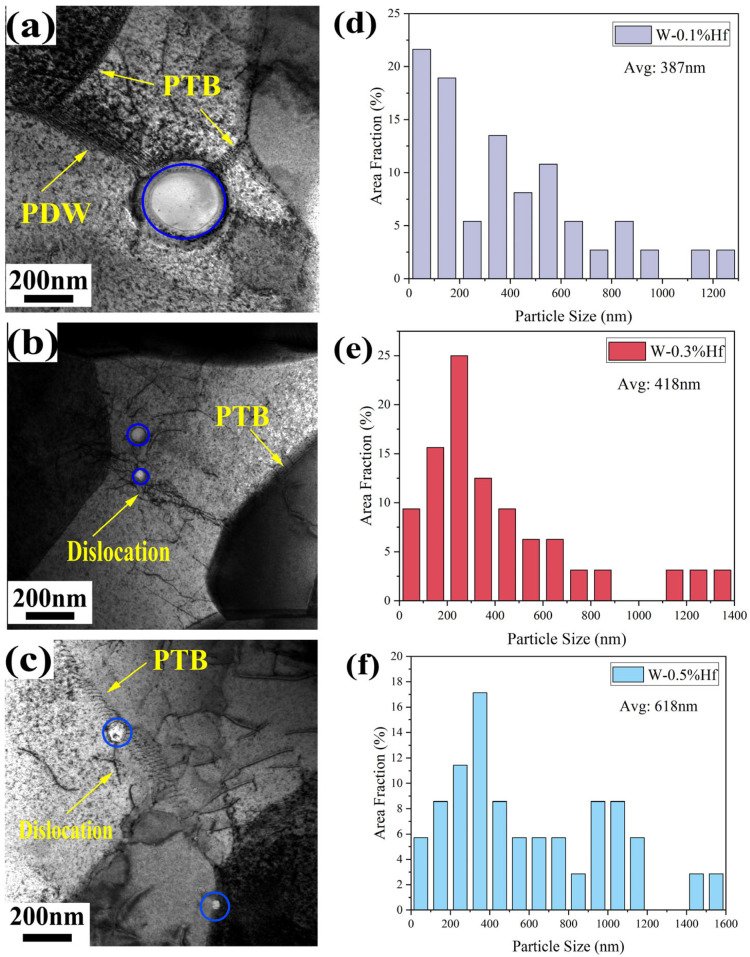
(**a**–**c**) Second-phase nanoparticles (in the blue circle) tightly bound to the GBs, dislocations interacting with intragranular second-phase nanoparticles, and TEM images showing the formation of fine tungsten grain from PDWs transforming to PTBs during the hot rolling process. The size distribution fractions of the second-phase particles of (**d**). W-0.1% Hf, (**e**). W-0.3% Hf, and (**f**). W-0.5% Hf, respectively.

The internal microstructure of the material was further analyzed by TEM images of W-0.3% Hf samples. As shown in Figure 3a–c, it can be observed that there are a large number of partially transformed boundaries (PTBs) and polygonal dislocation walls (PDWs) in the microstructure of the materials after hot rolling. This grain structure is achieved by strictly controlling the deformation, dynamic recovery, and dynamic recrystallization processes. In addition, it can be also observed that the second-phase particles mainly have two distribution positions. One is distributed inside the grain, and the second-phase nanoparticles with pinned dislocations are in the grain. The other is the second-phase particles with larger particle size at the grain boundary. The distribution uniformity and particle size of the dispersed second phase directly determine the properties of the W-Hf materials. According to the TEM images, the number density of the dispersed second phase and the size of nearly 50 s phase nanoparticles in the alloy with different Hf content were counted, as shown in Figure 3d–f. For W-0.1% Hf alloy, the number density of the second-phase particles is 3.3 × 10^17^ m^−3^, the number density of the second-phase particles in W-0.3% Hf alloy is 6.4 × 10^17^ m^−3^, and it is noteworthy that 71.8% of the second-phase particles are smaller than 500 nm, as shown in Table 2. For W-0.5% Hf alloy, although the amount of Hf added has increased, the number density of the second phase is only 2.8 × 10^17^ m^−3^. This is due to the agglomeration of the second-phase particles, and the merger and growth of the particles during sintering and hot rolling. Even nearly 28.5% of the large second-phase particles with a particle size of more than 1 μm are agglomerated at the grain boundary, and the strengthening effect is not ideal.

**Figure 4:**
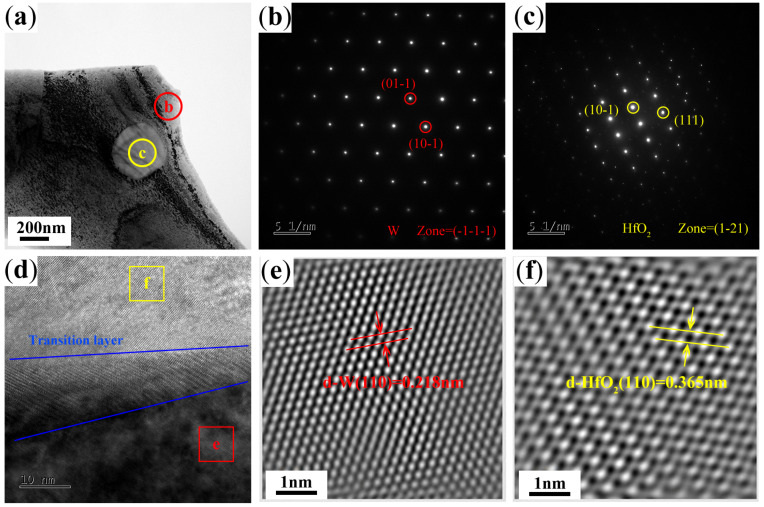
Detailed analysis of the structure of W matrix and second-phase particles. (**a**) TEM images. (**b**) The SAEDP revealing the W matrix (b in (**a**) with a body centered cubic structure). (**c**) The SAEDP revealing the second particle (c in (**a**) with a monoclinic structure). (**d**) HRTEM image of W matrix and HfO_2_ phase (intragranular) as viewed along [001]. (**e**) Inverse fast Fourier transform (IFFT) pattern of selected red square area e on W matrix. (**f**) IFFT pattern of selected yellow square area f on the second particle.

The microstructure of the second phase was further analyzed by adding W-0.5% Hf alloy with more Hf content. Figure 4a is the bright field image of the TEM morphology of the spherical second-phase particles dispersed in the tungsten grain with a diameter of about 300 nm. The EDS spectrum of the second-phase particles shows that the particle composition is W, Hf, and O, and the element mass ratio is 13:71:16. The crystal structure of the matrix phase and the second phase was analyzed by electron diffraction. As shown in Figure 4b,c, the matrix phase is a W phase with an obvious bcc structure, and the second-phase particles have a monoclinic phase structure. The high-resolution projection electron microscopy images of the interface between the second phase and the matrix were further analyzed along the [001] direction, as shown in Figure 4d, and the high-resolution inverse fast Fourier transform images of the matrix phase and the second phase were obtained, as shown in Figure 4e,f. Through calibration, it can be determined that the particles pinned inside the tungsten grain are HfO_2_ particles with monoclinic crystal structure. In order to eliminate accidental results, multiple nanoparticles pinned inside the tungsten grains or on the tungsten grain boundaries were selected for energy spectrum analysis, and the presence of O elements was found. In the actual sintering process, the impurity elements at the W grain boundary are mainly the presence of O. After the decomposition of HfH_2_ powder, the active elemental Hf is easily combined with the impurity O to form stable HfO_2_ particles, which pins the grain boundary and hinders the movement of the grain boundary. In addition, in the interior of the grain, a small part of the retained HfH_2_ powder decomposes to form a very fine solution, and only a few tens of nano-sized elemental Hf particles also combine with the matrix W to form a small amount of fine Hf-W solid solution or intermediate phase HfW_2_, and then combine with the impurity O diffused into the interior of the grain boundary to precipitate more stable HfO_2_ particles or W-Hf-O particles at a high temperature. The sizes of the second-phase particles precipitated in this part are smaller, and the precipitation strengthening effect is better. Similar results were found in a study on other active elements strengthening W-based materials [29].

### 3.3. Mechanical Properties of the PW and W-Hf Materials

**Figure 5:**
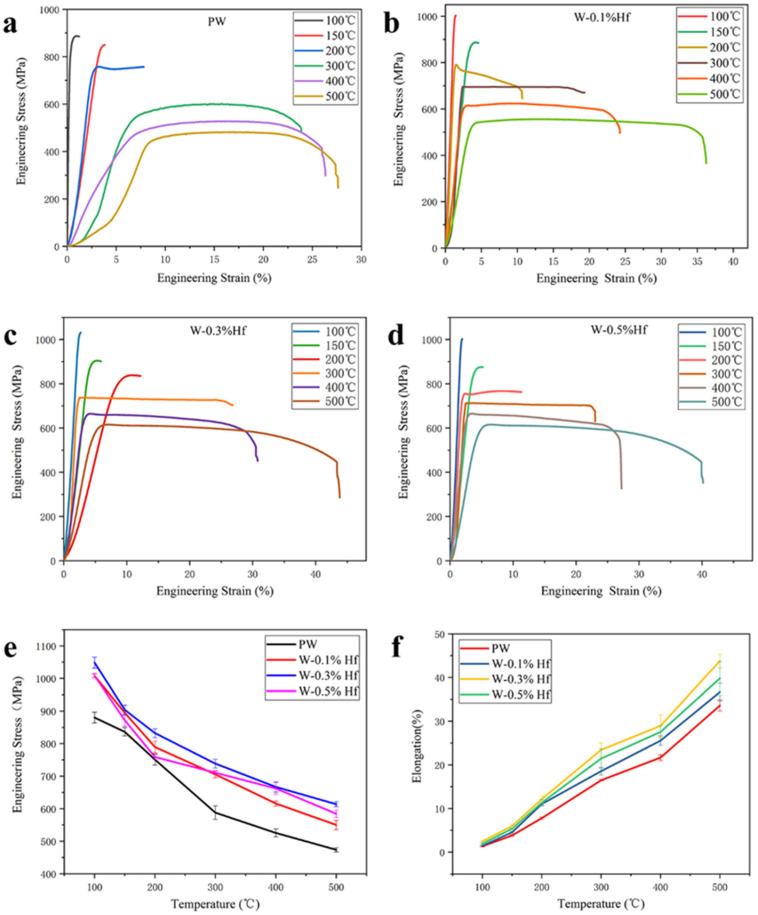
Engineering stress–strain curves of PW and W-Hf samples tested at different temperatures during tensile test. (**a**) PW samples; (**b**) W-0.1% Hf; (**c**) W-0.3% Hf; (**d**) W-0.5% Hf. (**e**) Summary of ultimate tensile strength (UTS) of different samples at different temperatures. (**f**) Summary of percentage elongation of different samples at different temperatures.

The high temperature tensile tests of PW and R-WHf materials were carried out in a vacuum atmosphere at a temperature range of 100–500 °C. Figure 5 shows that the pure tungsten sample and W-0.1% Hf still exhibit brittle fracture at 150 °C, and the total elongations are 3.87 ± 0.17% and 4.61 ± 0.18%, respectively. At 200 °C, these two alloys exhibit ductile fractures, and the total elongations are 7.83 ± 0.24% and 10.66 ± 0.34%, respectively. If the minimum temperature at which the total elongation of the sample exceeds 5% without fracture is defined as DBTT [27], it indicates that the DBTT of PW and W-0.1% Hf samples is about 200 °C in this work. The W-0.3% Hf and W-0.5% Hf samples show a ductile fracture at 150 °C, with a total elongation of 5.91 ± 0.41% and 5.34 ± 0.11%, respectively. The DBTT of W-0.3% Hf and W-0.5% Hf is about 150 °C, which is better than other values reported in the literature [30]. The UTS of the three contents of W-Hf at 100 °C is greater than 1000 MPa; in particular, the UTS of the W-0.3% Hf sample, which is up to 1048 ± 17.01 MPa. Under the tensile test at 500 °C, the corresponding UTS is 614 ± 7.55 MPa, and the total elongation is as high as 43.77 ± 1.54%. At the same test temperature, the UTS and total elongation of the W-0.3% Hf sample are better than those of pure tungsten and other composition alloys, showing the most excellent mechanical properties. The improvement of strength and toughness of W-based materials is due to the fine grain strengthening and the dispersion strengthening of the HfO_2_ s phase. The smaller the second-phase particle size is, the higher the number density is and the better the dispersion strengthening effect is [31]. As the temperature of the tensile test increases, all samples undergo a transition from brittle fracture to ductile fracture, and the UTS also decreases with increasing temperature, as shown in Figure 5e,f. This is because the high temperature increases the diffusion capacity of the atoms, resulting in an increase in vacancy atoms and a change in the slip system, making plastic deformation more likely to occur.

**Figure 6:**
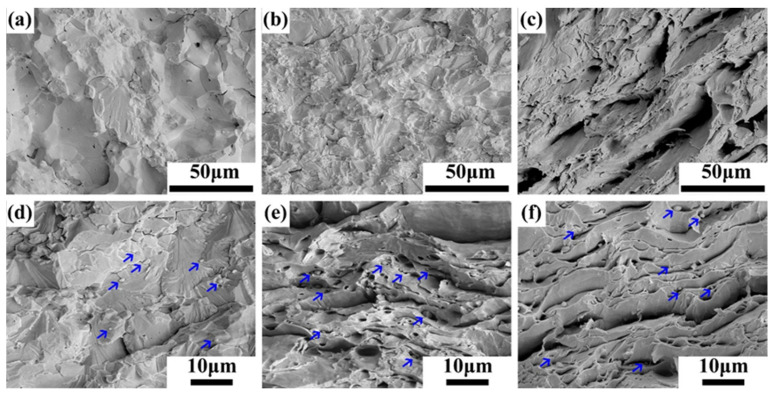
Microstructure of the tensile test fracture at different temperatures. (**a**) PW at 150 °C. (**b**) PW at 300 °C. (**c**) PW at 500 °C. (**d**) W-0.3 wt% Hf at 150 °C. (**e**) W-0.3 wt% Hf at 300 °C. (**f**) W-0.3 wt% Hf at 500 °C. The blue arrows in (**d**–**f**) indicate that the second-phase particles in the W-0.3% Hf alloy are distributed in the fracture.

Figure 6 is the tensile fracture morphology of pure tungsten and W-0.3% Hf alloy at different temperatures, and it is worth noting that the blue arrows in Figure 6d–f indicate that the second-phase particles are distributed in the fracture. At 150 °C, the fracture of the pure W sample shows obvious intergranular brittle fracture characteristics, including only a small part of the transgranular cleavage fracture, as shown in Figure 6a, while the fracture of the W-0.3% Hf sample at the same temperature is dominated by the transgranular cleavage fracture, the intergranular fracture basically disappears, and the crack can be observed to terminate in the second-phase particles. As shown in Figure 6d, the second-phase particles can hinder crack propagation during plastic deformation and increase the energy required for fracture to form a new surface. In addition, due to the smaller grain size of W-0.3% Hf sample, the grain boundary density increases, the dislocation pile-up and stress concentration decrease, and the mechanical properties of the alloy are further improved. When the tensile temperature increases to 300 °C, as shown in Figure 6b,d, the intergranular fracture of pure W basically disappears, but the W-0.3% Hf sample has obvious ductile fracture characteristics, and there are a large number of dimples. In the tensile fracture at 500 °C, both samples showed obvious dimple structure, but compared with pure W, the number of W-0.3% Hf dimples increased significantly, and a large number of second phases were observed in the center of the dimples. In addition, it can be observed that the grains undergo obvious drawing deformation under stress. 

### 3.4. Recrystallization of the PW and W-Hf Alloys

**Figure 7:**
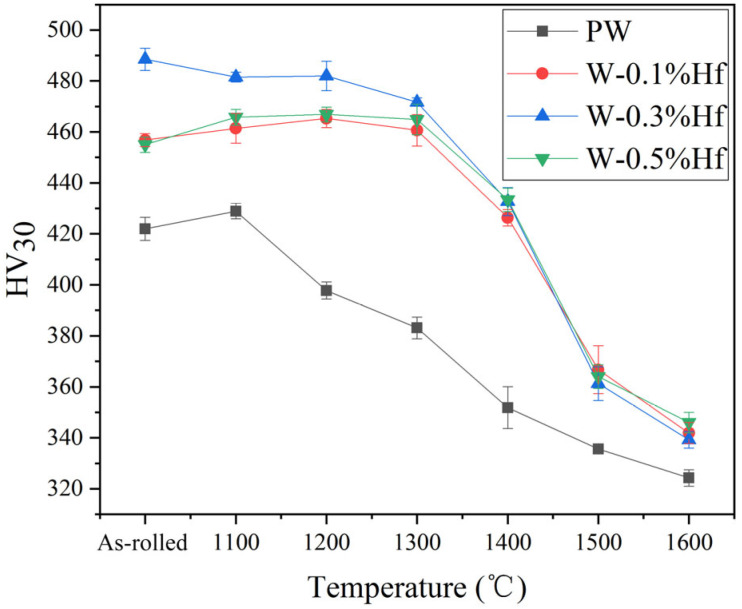
Effect of annealing temperature on the HV_30_ of PW and W-Hf alloys.

Figure 7 shows the change in the hardness of PW and W-Hf alloys in their original rolling state and after annealing at different temperatures for 1 h. For the PW, the Vickers hardness is 428.9 ± 3.00 HV_30_ after annealing at 1100 °C. This process releases the residual stress, and the hardness change is not significant compared with the original rolled sheet (421.93 ± 4.53 HV_30_). When the annealing temperature rises to 1200 °C, the hardness decreases sharply to 397.8 ± 3.36 HV_30_, indicating that the material has recrystallized. The decrease in hardness is due to the decrease in dislocation density during recrystallization, resulting in new grains without distortion [32]. Therefore, the recrystallization temperature of PW is determined to be 1200 °C. For W-Hf alloys, in the annealing temperature range of 1100–1300 °C, the hardness change curves of the three components all show a plateau period, and the Vickers hardness fluctuates slightly. However, when the annealing temperature was 1400 °C, the Vickers hardness of W-0.1% Hf decreased to 426.33 ± 3.18 HV_30_, the Vickers hardness of W-0.3% Hf decreased to 432.66 ± 5.48 HV_30_, and the Vickers hardness of W-0.5% Hf decreased to 433.36 ± 4.57 HV_30_. The hardness change curves all decreased sharply, indicating that the recrystallization temperature of W-Hf alloy samples was about 1400 °C. In this work, the amount of Hf added did not cause significant difference in the recrystallization temperature of W-Hf alloy. Compared with the rolled pure tungsten sample in the same preparation process, the recrystallization temperature of the W-Hf alloy sample increased by more than 200 °C.

**Figure 8:**
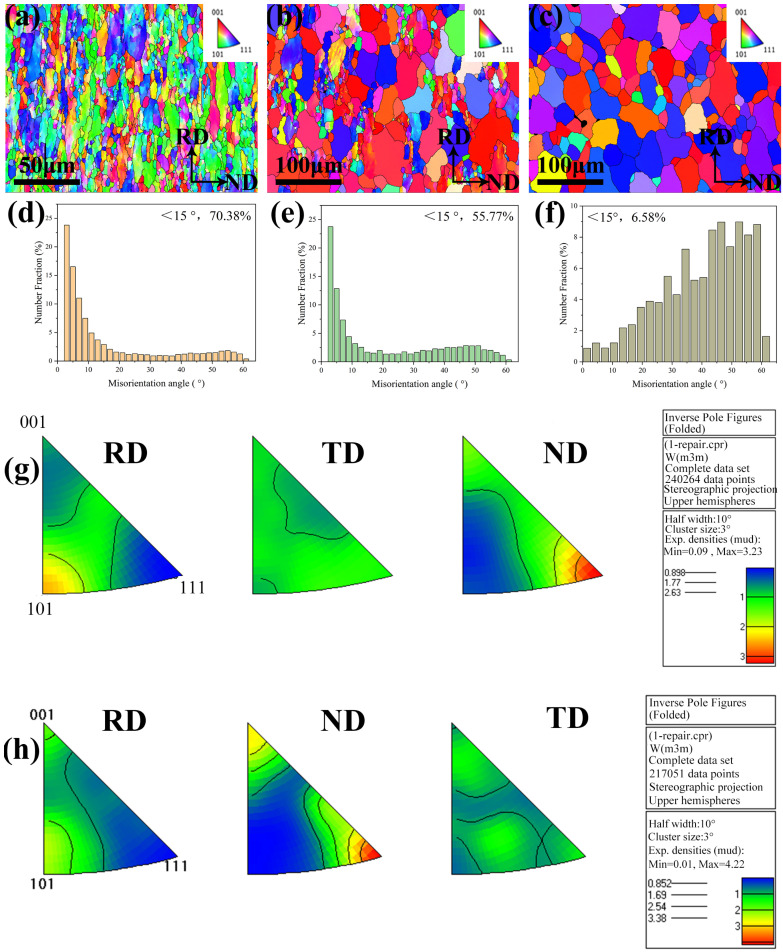
EBSD orientation maps of RD-ND surface after annealing at 1200 °C (**a**), 1400 °C (**b**), and 1600 °C (**c**) for 1 h. Grain boundary misorientation distribution maps after annealing at 1200 °C (**d**), 1400 °C (**e**), and 1600 °C (**f**) for 1 h. IPF after annealing at 1200 °C (**g**) and 1600 °C (**h**) for 1 h.

Figure 8 shows the EBSD mapping, orientation difference distribution map and inverse pole figure (IPF) of W-0.3% Hf alloy RD-ND surface after annealing at 1200 °C, 1400 °C, and 1600 °C. The corresponding relationship between color and orientation is as follows: red for <001>, green for <110>, and blue for <111>. Figure 8a,d show that after annealing at 1200 °C, the alloy still exhibits obvious slender grains. The grain boundaries in the alloy are mainly small angle grain boundaries, accounting for 70.38%, and the orientation difference distribution still deviates from the Mackenzie distribution curve. After annealing at 1400 °C, as shown in Figure 8b,e, the alloy began to appear equiaxed grains after recrystallization, and the low-angle grain boundaries began to disappear, accounting for 55.77%. For the fully recrystallized sample at 1600 °C, it is mainly composed of undistorted equiaxed grains, and its orientation difference distribution is closer to the Mackenzie distribution baseline. Figure 8g,h show that the rolled samples are mainly composed of <110>//RD and <111>//ND textures at 1200 °C annealing temperature. After complete recrystallization, the <110>//RD strength decreases, while the <111>//ND strength has almost no significant change.

## 4. Discussion

**Figure 9:**
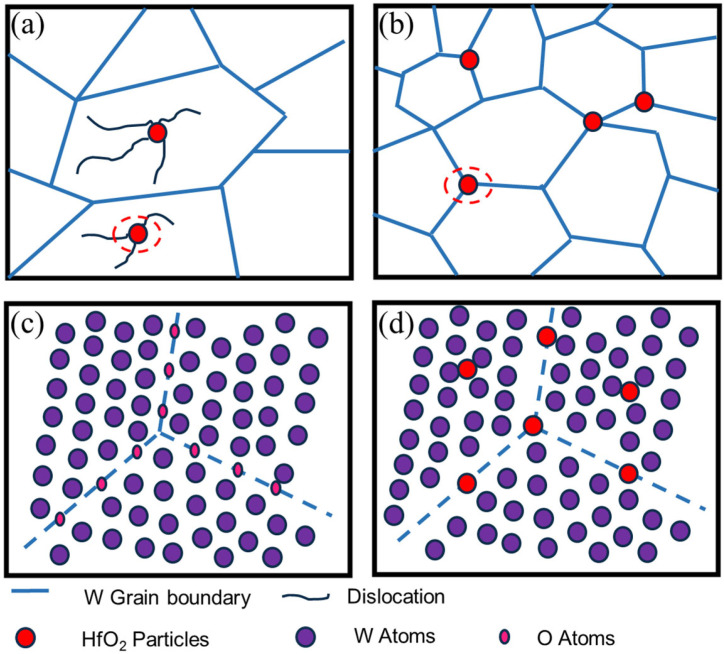
Schematic diagram of the effect of Hf addition. (**a**) Intragranular second-phase nanoparticles can pin and accumulate dislocations inside the grain. (**b**) Second-phase particles that are tightly bound to the GBs can impede GB sliding. (**c**) Impurity elements distributed at the grain boundaries of pure tungsten. (**d**) Formation process of second-phase particles of HfO_2_.

In this section, the strengthening and toughening mechanism of the W-Hf alloy is analyzed through the microstructure of W-Hf alloy, as shown in Figure 9. Firstly, through strictly controlled multi-pass hot rolling process, deformation, dynamic recovery, and dynamic recrystallization occur simultaneously. With the increase in rolling deformation, the W grain boundary as a dislocation source produces a large number of dislocations, high-density dislocations react under the drive of stress and temperature, and dislocation tangles form low-energy dislocation walls and cellular substructures. Due to the principle of minimum temperature driving force and energy, dislocation cells and dislocation walls will eventually form fine equiaxed sub-grains and a large number of sub-grain boundaries. In contrast, hot rolling with strict control of the processing route is very different from conventional hot rolling and cold rolling. The cold rolling processing route leads to grain elongation, and the grains have obvious anisotropy and instability [33]. The average size of sub-grains in W-0.3% Hf alloy is only 4.32 μm. The widespread existence of equiaxed sub-grains increases the grain boundary density, reduces the anisotropy of grain orientation, and hinders dislocation slip and crack propagation [34]. On the other hand, refined grains can greatly increase the area of grain boundaries and reduce impurities at grain boundaries, thus weakening the embrittlement effect of impurities on grain boundaries and reducing the DBTT of tungsten alloys. Secondly, the active Hf element obtained by the decomposition of HfH_2_ will absorb the impurity elements segregated at the W grain boundary, purify the grain boundary and improve the grain boundary binding force. In addition, the second phase HfO_2_ particles formed will pin onto the grain boundary, hinder the grain growth, and refine the grain, thus improving the mechanical properties of pure tungsten. Thirdly, elemental Hf with a fine particle size that remaining in the W grains is dissolved in the W matrix and combined with the diffused impurity O. Only a few dozen nanoscale HfO_2_ s phase particles were precipitated, which can pin and aggregate dislocations in the tungsten grains, so that the dislocations are distributed in the grains rather than piled up at the grain boundaries to cause stress concentration. This dislocation pinning greatly improves the strength and toughness of the material.

## 5. Conclusions

In this paper, large-sized W-(0, 0.1, 0.3, 0.5) % Hf bulk materials were prepared by a powder metallurgy process combined with high temperature rolling deformation. The morphology and microstructure of the materials with different compositions were characterized, and the high temperature mechanical properties were measured. The following conclusions were drawn:

(1) The successfully prepared W-Hf alloy provides a new development idea for future PFMs. Hf-active elements introduced by HfH_2_ powder can be combined with impurity O in W grains to purify and strengthen grain boundaries. The formed second phase HfO_2_ particles can refine grains and form dispersion strengthening, pinning dislocations and grain boundaries. In addition, the hot rolling process is strictly regulated. This manufacturing route is suitable for the mass production of tungsten alloys for engineering applications. It can form a wide range of equiaxed sub-grain microstructures, reduce the anisotropy of grain orientation, and is conducive to the synergistic improvement of material strength and toughness.
(2) The number density of the second phase in W-0.3% Hf alloy is 6.4 × 10^17^ m^−3^, and 71.8% of the second-phase particles are smaller than 500 nm. The second phase is small and dispersed, so it has excellent toughness and strength. At 100 °C, the UTS is 1048 ± 17.02 MPa. At 150 °C, it shows ductile fracture, and the total elongation is 5.91 ± 0.41%. Under the tensile test at 500 °C, the corresponding ultimate tensile strength is 614 ± 7.55 MPa, and the elongation is as high as 43.77 ± 1.54%.
(3) The high temperature stability of pure tungsten and W-Hf alloys was studied by isothermal annealing experiments. The recrystallization temperature of W-Hf alloys is 1400 °C, which is 200 °C higher than that of pure tungsten. At the same time, the complete recrystallization of W-0.3% Hf was observed after annealing at 1600 °C, accompanied by the disappearance of the RD//<110> texture.

## Figures and Tables

**Table 1:** Summary of room-temperature properties of hot-rolled PW and W-Hf alloys.

	True Density g/cm^3^	Relative Density (%)	RD-TD Surface HV_30_	TD-ND Surface HV_30_
PW	19.09 ± 0.01	99.16 ± 0.06	431.95 ± 3.74	436.2 ± 2.56
W-0.1% Hf	19.09 ± 0.02	99.27 ± 0.11	463.48 ± 6.38	464.7 ± 3.68
W-0.3% Hf	19.08 ± 0.01	99.43 ± 0.03	481.47 ± 4.82	476.5 ± 1.95
W-0.5% Hf	18.99 ± 0.02	99.19 ± 0.09	464.87 ± 5.79	456.2 ± 4.32

**Table 2:** Size distribution of second-phase particles in alloys with different compositions.

	W-0.1% Hf (%)	W-0.3% Hf (%)	W-0.5% Hf (%)
0–200 nm	40.5	23.4	14.2
200–500 nm	27.1	48.4	37.2
500 nm-1 μm	27.0	18.9	20.1
>1 μm	5.4	9.3	28.5

## Data Availability

The data are contained within the article.
